# Connexin 30 deletion exacerbates cochlear senescence and age-related hearing loss

**DOI:** 10.3389/fcell.2022.950837

**Published:** 2022-08-09

**Authors:** Fabiola Paciello, Veronica Zorzi, Marcello Raspa, Ferdinando Scavizzi, Claudio Grassi, Fabio Mammano, Anna Rita Fetoni

**Affiliations:** ^1^ Department of Neuroscience, Università Cattolica del Sacro Cuore, Rome, Italy; ^2^ Fondazione Policlinico Universitario A. Gemelli IRCCS, Rome, Italy; ^3^ CNR Institute of Biochemistry and Cell Biology, Monterotondo (RM), Italy; ^4^ Department of Physics and Astronomy, University of Padova, Padova, Italy; ^5^ Department of Otolaryngology Head and Neck Surgery, Università Cattolica del Sacro Cuore, Rome, Italy; ^6^ Unit of Audiology, Department of Neuroscience, Reproductive Sciences and Dentistry, University of Naples Federico II, Naples, Italy

**Keywords:** connexins, inner ear, inflammation, oxidative stress, vascular dysfunction, presbycusis, genetic risk factors

## Abstract

Pathogenic mutations in the Gjb2 and Gjb6 genes, encoding connexin 26 (Cx26) and connexin 30 (Cx30), respectively, have been linked to the most frequent monogenic hearing impairment, nonsyndromic hearing loss, and deafness DFNB1. It is known that Cx26 plays an important role in auditory development, while the role of Cx30 in hearing remains controversial. Previous studies found that partial deletion of Cx26 can accelerate age-related hearing loss (ARHL), a multifactorial complex disorder, with both environmental and genetic factors contributing to the etiology of the disease. Here, we investigated the role of Cx30 in cochlear-aging processes using a transgenic mouse model with total deletion of Cx30 (Cx30 ΔΔ mice), in which Cx30 was removed without perturbing the surrounding sequences. We show that these mice are affected by exacerbated ARHL, with increased morphological cochlear damage, oxidative stress, inflammation, and vascular dysfunctions. Overall, our data demonstrate that Cx30 deletion can be considered a genetic risk factor for ARHL, making cochlear structures more susceptible to aging processes.

## 1 Introduction

Connexin 30 (Cx30) and connexin 26 (Cx26), which co-assemble in gap junction (GJ) channels, are the principal GJ proteins in the developing and mature mammalian cochlea (Mammano, 2019). These proteins play a key role in cochlear physiology, are responsible for the direct passage of ions and small molecules between the cytoplasm of cochlear nonsensory cells, and are considered crucial for hearing (Forge et al., 2003; Martínez et al., 2009). Indeed, pathogenic mutations in the DFNB1 locus, which contains both genes (GJB2/Cx26 and GJB6/Cx30) encoding these connexins, are the most frequent cause of monogenic inheritance for nonsyndromic prelingual deafness (Snoeckx et al., 2005; Kenna et al., 2010; del Castillo and del Castillo, 2017). Even though the crucial role of GJB2 and GJB6 variants in determining hearing loss is well known and supported by clinical and experimental evidence, less is known about the physiological function of inner ear connexins, and how they contribute to the etiopathogenesis of deafness remains still elusive.

Experimental evidence, based on the use of knock-out (KO) mouse models, confirmed the essential role of inner ear connexins for hearing, since their absence causes profound deafness associated with apoptotic processes in the organ of Corti during development (Cohen-Salmon et al., 2002; Teubner, 2003; Crispino et al., 2011; Johnson et al., 2017; Fetoni et al., 2018). In our previous studies, we showed that homozygous offsprings with targeted deletion of Cx26 in the epithelial GJ network of the cochlea (Gjb2^−/−^ mice) failed to acquire hearing (Crispino et al., 2011; Johnson et al., 2017) and their heterozygous (Gjb2+/−) siblings are affected by accelerated presbycusis, also known as age-related hearing loss (ARHL; Fetoni et al., 2018).

In this study, we focused on the effect of Cx30 total deletion on hearing function during aging. Cx30 inactivation causes profound deafness (Teubner, 2003; Cohen-Salmon et al., 2007; Sun et al., 2009; Chen et al., 2022), but in some cases, it strongly reduces the expression of Cx26 (Ortolano et al., 2008; Lynn et al., 2011). Overexpression of Cx26 in Cx30 KO mice rescued hearing (Ahmad et al., 2007), but not vice versa (Qu et al., 2012). Here, we used the Cx30 ΔΔ mouse model developed by Boulay et al. (2013). In contrast to the previous model (Teubner, 2003), in Cx30 ΔΔ mice, the Gjb6 gene (Cx30) was deleted with reduced perturbation of the surrounding sequences, leading to a less dramatic impact on the expression of the nearby Gjb2 gene that encodes Cx26. Although Cx30 ΔΔ mice show normal hearing (Boulay et a, 2013), we wondered whether Cx30 deletion could increase cochlear susceptibility to aging processes and whether Gjb6 deletion can be considered a genetic risk factor for ARHL. To address these issues, we compared the hearing function of Cx30 ΔΔ mice and their wild-type (WT) siblings up to 12 months of age (MoA). Our results support the role of Gjb6 in cochlear-aging, given that its deletion can exacerbate ARHL making cochlear structures more vulnerable to age-induced oxidative stress, causing inflammation and vascular damage.

## 2 Materials and methods

### 2.1 Animal model

Animals were bred and genotyped in the Consiglio Nazionale delle Ricerche (CNR) Monterotondo node of the European Mouse Mutant Archive (EMMA) (Raess et al., 2016), an ESFRI/INFRAFRONTIER Distributed Research Infrastructure (https://www.infrafrontier.eu/). Animals were housed individually in ventilated caging systems (Tecniplast, Gazzada, Italy) at controlled temperature (21°C ± 2°C) and humidity (55 ± 15%) with 50–70 air changes per hour and under a controlled (12:12 h) light–dark cycle (7 a.m.−7 p.m.). Access to water was *ad libitum* and a standard rodent diet was administered (Emma 23, Mucedola, Settimo Milanese, Italy). Cx30 ΔΔ mice are on a C57BL/6 background and were obtained by crossing Cx30fl/fl mice with Pgk-Cre mice, as described in Boulay et al. (2013). Genotyping protocols were performed by PCR using the primers previously described (Boulay et al., 2013). In particular, genotyping of the Cx30Δ allele was performed using the Gjb6F 5′-GCA​GTA​ACT​TAT​TGA​AAC​CCT​TCA​CCT-3′ and a primer binding downstream of the third loxP site Gjb6ΔR 5′-CCC​ACC​ATC​AAG​GTT​GAA​CT-3′.

### 2.2 Measurement of the auditory brainstem response

To assess mouse auditory function, we recorded auditory brainstem responses (ABRs) using a dedicated Workstation (Tucker-Davis Technologies, Inc., Alachua, FL, U.S.A.). Mice were anesthetized with an intraperitoneal injection of ketamine (70 mg/g for males and 100 mg/g for females) and medetomidine (1 mg/g). The body temperature was maintained at 37°C using a heating pad under feedback control. Animals were placed in a sound-attenuating cabin (ETS-Lindgren SD Test Enclosure, MDL Technologies Limited, Hitchin, United Kingdom). Acoustic stimuli were clicks (100 μsec duration) and 4, 8, 16, 24, and 32 kHz tone bursts (1 ms rise–fall time with 3 ms plateau), delivered in the free field using an MF1-M speaker. Needle electrodes (27 gauge, 13 mm; Cat. No. S83018-R9, Rochester) were inserted subcutaneously at the vertex (active), ventrolateral to the left ear (reference), and above the tail (ground) to collect bioelectrical potentials. Responses were amplified, filtered (0.3–3 kHz), and averaged over 512 presentations of the same stimulus. Hearing threshold levels were determined offline as the SPL at which a Wave I peak could be visually identified above the noise floor (0.1 μV). ABRs were recorded at different ages (2, 6, and 12 MoA).

### 2.3 Morphological analysis: Hair cells and spiral ganglion neuron count

#### 2.3.1 F-actin staining

To evaluate hair cell survival at 12 MoA, 4 cochleae/group were used from Cx30 WT and Cx30 ΔΔ animals. Cochleae were quickly removed and fixed with 4% paraformaldehyde. After tissue dissection, the basilar membrane was isolated and F-actin staining was performed by incubating samples with ActinGreen 488 Ready Probes Reagent (ThermoFisher, Cat. No. R37110). After washing with PBS, the samples were mounted onto glass slides with a mounting medium (FluorSaveTM Reagent, Merk, Cat. No. 345789) and analyzed using a confocal microscope (TCS SP5, Leica) equipped with an oil-immersion objective (40× HCX PL APO 1.25 N.A., Leica).

#### 2.3.2 Hematoxylin-eosin staining

To determine the spiral ganglion neuron (SGN) density at 12 MoA, 4 cochleae/group were used. Cochleae were quickly removed, and the samples were fixed with 4% paraformaldehyde in PBS at 4°C and a pH of 7.5. Next, we incubated samples for 3 days in 10% ethylenediaminetetraacetic acid (EDTA). Then, after decalcification, samples were incubated for 48 h in sucrose (30%), included in OCT (Killik, Bio-Optica, Milan, Italy), and longitudinal sections were cut at a thickness of 6 μm using a cryostat (CM 1950; SLEE medical GmbH, Mainz, Germany). Morphological analysis of SGNs was performed by using hematoxylin and eosin (H&E) staining on slides, to detect viable and nonviable stained cells. A standard H&E protocol was used with 4–5 min incubation in hematoxylin and 45 s staining in eosin. Slides were then mounted with Entellan^®^ (Merck) and the images were captured using an Olympus BX63 microscope. Viable neurons with a clear round nucleus and homogeneous cytoplasm were then counted. The SGN density (cells per square millimeter) was calculated using NIH ImageJ 1.43u (Image Processing and Analysis in Java).

### 2.4 Immunohistochemistry and confocal imaging

At 12 MoA, after the last ABR recording, animals were sacrificed with a lethal dose of anesthesia (ketamine: 70 mg/g for males and 100 mg/g for females and medetomidine: 1 mg/g); the cochleae were quickly removed, fixed with 4% paraformaldehyde in PBS at 4°C and a pH of 7.5, and processed as described hereafter.

To perform immunofluorescence analyses, cochleae from Cx30 WT and Cx30 ΔΔ mice were decalcified by incubating the samples in EDTA, as described above. Then, specimens were: 1) included in 3% agarose dissolved in PBS and cut to obtain longitudinal sections at a thickness of 100 μm using a vibratome (VT 1000 S, Leica) or 2) included in OCT and cryosectioned to obtain 12 μm longitudinal sections (Cryostat CM 1950; SLEE). Permeabilization of tissues was performed by incubating the specimens in a solution containing 0.1% Triton X-100 and 2% bovine serum albumin in PBS. Sections were then incubated overnight at 4°C with the following primary antibodies: mouse monoclonal Cx26 (diluted 1:100 in PBS, ThermoFisher, Cat. No. 335800), Cx30 (diluted 1:100 in PBS, ThermoFisher, Cat. No. 71–2,200), and rabbit polyclonal VEGF-C (diluted 1:100 in PBS, Santa Cruz Tech., Cat. No. sc-9047). Secondary antibodies (Alexa Fluor^®^ 488 goat antimouse IgG, ThermoFisher, Cat. No. A11029 or Alexa Fluor^®^ 546 donkey antirabbit IgG, ThermoFisher, Cat. No. A10040) diluted 1:400 in PBS were applied at room temperature (RT, 22°C–25°C). AlexaFluor 568 phalloidin (1 U/ml, ThermoFisher, Cat. No. A12380) and 4′,6-diamidino-2-phenylindole (ThermoFisher, Cat. No. D1306) (1:1,000) were used to label F-actin and cell nuclei respectively. The sections were then coverslipped with an antifade medium (FluorSave™ Reagent, Merk). To assess the integrity of the cochlear blood barrier, the stria vascularis was isolated after cochlear dissection as described by Gentile et al. (2021) and the samples were incubated with Alexa Fluor^®^ 546 donkey antimouse IgG (ThermoFisher) for 2 h at RT. For all immunofluorescence analyses, control experiments were performed. Staining was absent in cochlear samples used as the negative control, obtained by omitting the primary antibody during the processing of tissues randomly selected across experimental groups, thus showing the absence of spontaneous or nonspecific fluorescence signal (data not shown). Images were acquired using a confocal microscope system (Nikon Ti-E, Confocal Head A1 MP, Tokyo, Japan or TCS SP5, Leica, Wetzlar, Germany).

#### 2.4.1 Dihydroethidium assay

We characterized the level of oxidative stress in cochlear samples, by analyzing reactive oxygen species (ROS) amount through a dihydroethidium (DHE) assay. DHE is a lipophilic cell-permeable dye that, in the presence of free radicals, is rapidly oxidized to ethidium. The produced ethidium is fixed by intercalation into nDNA, so that it gives an indication of oxidative stress status.

Cochlear specimens (4 cochleae/group) were then incubated with 1 μM DHE (Invitrogen, Carlsbad, CA, USA, Cat. No. D23107) in PBS for 30 min at 37°C and then coverslipped with an antifade medium (FluorSave™ Reagent). Images were obtained by using two-photon excitation (792 nm, <140 fs, 90 MHz) performed using an ultrafast tunable mode-locked titanium:sapphire laser (Chameleon, Coherent) and a confocal microscopy system (Nikon).

### 2.5 Western immunoblot

To perform western blot analyses, cochleae of Cx30 WT and Cx30 ΔΔ animals (8 cochleae/group) at 12 MoA were removed, collected on ice, stored at –80°C, and homogenized to extract total protein levels by using ice-cold RIPA buffer (Pierce) containing 50 mM Tris, 150 mM NaCl, 1 mM EDTA, 1% DOC, 1% Triton X-100, 0.1% SDS, and 1 ×  protease, phosphatase-1, and phosphatase-2 inhibitor cocktails (Sigma). The lysate was sonicated 3 times at 10 Hz (Hielscher, Ultrasound Technology UP50H/UP100H) and centrifuged (13,000 rpm, 15 min, 4°C). The total protein concentration was then evaluated using the microBCA kit (Pierce). Then, we added a reducing sample buffer, and the samples were heated to 95°C for 5  min.

Protein lysates (70 μg) were loaded onto Tris–glycine polyacrylamide gels for electrophoretic separation. Colorburst™ Electrophoresis Markers (Sigma or BioRad) were used as molecular mass standards. Proteins were then transferred onto nitrocellulose membranes at 100 V for 2 h at 4°C in a transfer buffer containing 25 mM Tris, 192 mM glycine, 0.1% SDS, and 20% methanol. Membranes were incubated for 1  h with a blocking buffer (5% skim milk in TBST) and then incubated overnight at 4°C with the following primary antibodies: anti-Cx26 (ThermoFisher), anti-Cx30 (ThermoFisher), anti-HIF-1α (Cat. No. sc-53546, Santa Cruz Tech.), and anti-NFκ-B (Cell Signaling, Danvers, MA, USA, Cat. No. #8242). The membranes were then washed with TBST and incubated for 1  h at RT with HRP-conjugated mouse or rabbit secondary antibodies (Cell Signaling). To confirm equal protein loading under all experimental conditions, the membranes were then incubated with an anti-GAPDH (Abcam, Cat. No. 9485) or anti-α-tubulin mouse monoclonal antibody (Sigma-Aldrich, Cat. No. T6074). The membranes were then washed, and the bands were visualized with an enhanced chemiluminescence detection kit (GE Healthcare, Cardiff, UK, Cat. No. RPN2232). The protein expression was evaluated and documented using UVItec, Cambridge Alliance. Experiments were performed in triplicate.

### 2.6 Statistical analysis

The following statistical analyses were used: 1) Student’s two-tailed t-test for normally distributed data, by using a worksheet (Microsoft Office Excel 2017; Version 1.30) and 2) analysis of variance with posthoc comparison (Tukey’s test) using Statistica (version 6.0, Statsoft Inc.). The mean values are quoted ± standard error of the mean (s.e.m.). We considered *p*-values < 0.05 as significant.

## 3 Results

### 3.1 Lack of Cx30 exacerbates presbycusis in Cx30ΔΔ mice

**FIGURE 1:**
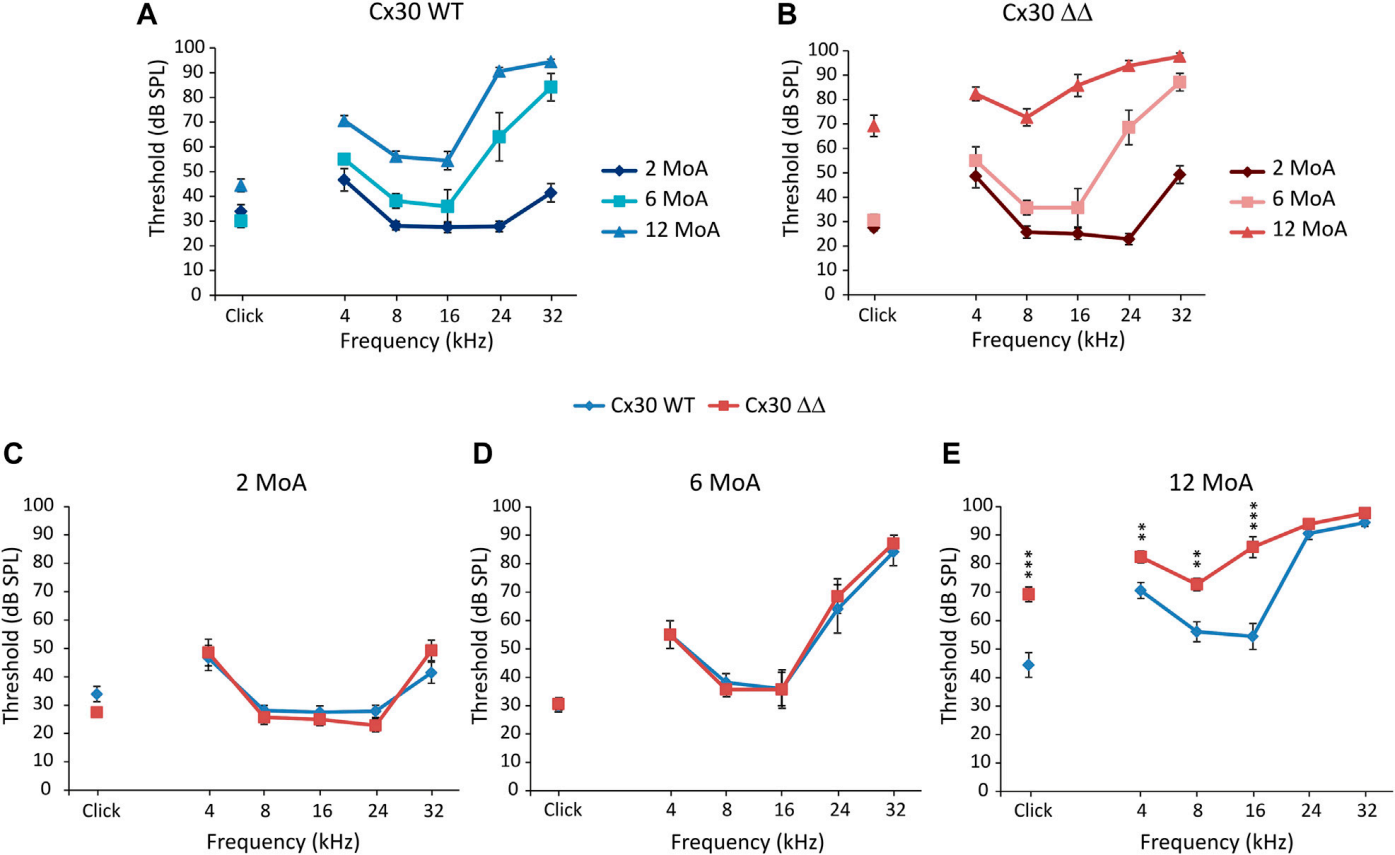
Connexin 30 deletion worsens ARHL. **(A and B)**: Graphs show *in vivo* auditory brainstem recordings (averaged threshold values ± standard error of the mean, S.E.M.) with click and tone burst responses in Cx30 WT **(A)** and Cx30 ΔΔ mice **(B)** at different months of age (MoA). For statistical significance, see Supplementary Table S1. **(C–E)**: Comparison of auditory thresholds between Cx30 WT (blue diamonds) and Cx30 ΔΔ (red squares) mice at 2 (A; Cx30 WT N = 9; Cx30 ΔΔ N = 8), 6 (B; Cx30 WT N = 11; Cx30 ΔΔ N = 7), and 12 (C; Cx30 WT N = 9; Cx30 ΔΔ N = 13) MoA. Asterisks indicate significant differences from three-way ANOVA (**p < 0.01 and ***p < 0.001).

To evaluate if Cx30 deletion affects hearing function, we assayed hearing performance by measuring ABRs to clicks and pure tone stimuli at various ages. Auditory thresholds did not differ between Cx30 WT and Cx30 ΔΔ mice at 2 MoA (Figures 1A–C). At 6 MoA, no differences in click responses were observed (Figure 1D) but threshold increase in both genotypes at high frequencies (24 and 32 kHz, Figures 1A, B, and D), as expected, according to the significant hearing loss for high frequencies (>20 kHz) was observed in C57BL/6 animals, showing early signs of presbycusis (Fetoni et al., 2011). A slight increase in hearing thresholds was observed in Cx30 WT animals at 12 MoA, compared to younger mice, and a severe hearing loss was still observed in the high-frequency region (Figure 1A). Aging affected auditory thresholds more severely in Cx30 ΔΔ mice (Figure 1B), with a significant worsening of hearing loss in mid and low frequencies and click responses, compared with age-matched WT animals (Figures 1A, B, and E). Thus, the absence of Cx30 in Cx30 ΔΔ mice causes a more severe hearing loss at 12 MoA, exacerbating presbycusis phenotype compared to the expected ARHL time course.

**FIGURE 2:**
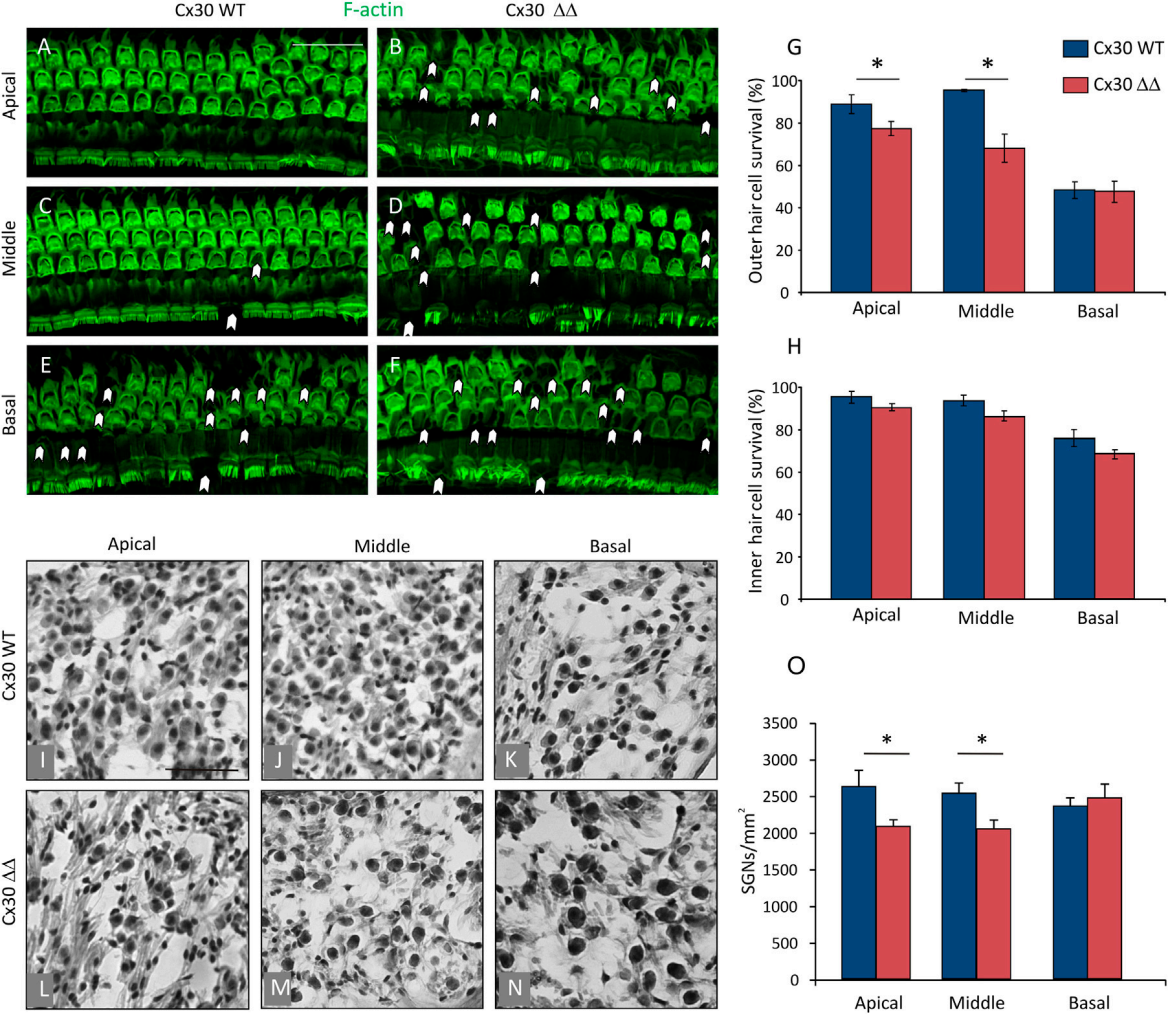
Connexin 30 deficiency exacerbates morphological cochlear damage induced by aging. **(A–F)**: Surface preparations of the basilar membrane with the organ of Corti from 12 MoA Cx30 WT and Cx30 ΔΔ mice; images from apical, medial, and basal turns were obtained by maximal intensity back projection of 15 confocal optical sections from a 0.8 µm step through-focus sequence (z-stack). Actin filaments were stained with ActinGreen 488 ReadyProbes (green). Arrowheads indicate missing cells. Scale bar: 50 μm. **(G and H)**: Histograms (mean ± S.E.M.) represent the percentage of OHC and IHC survival in the three cochlear turns from Cx30 WT and Cx30 ΔΔ animals. **(I–N)**: Representative SGN hematoxylin-eosin staining in Cx30 WT **(I–K)** and Cx30 ΔΔ samples **(I–N)** in apical, middle, and basal cochlear turns. **(O)**: Histograms (mean ± S.E.M.) represent the number of SGNs per mm^2^ in the three different cochlear turns. N = 4 cochleae/group. Asterisks indicate significant differences between groups from Student’s t-test (*p < 0.05).

To corroborate functional results at the morphological level, we focused on hair cell and SGN survival. Figure 2, upper panels, shows F-actin staining in surface preparations of the organ of Corti and outer (OHC) and inner (IHC) hair cell counts in Cx30 WT and Cx30 ΔΔ mice at 12 MoA. Consistent with functional data, in Cx30 WT animals (Figures 2E and G) of 12 MoA (Figures 2A, C, and E), a loss of OHC, characterized by dark spots, phalangeal scars, and disappearance of both cuticular plates and hair bundles (indicated by arrowheads), was observed in basal cochlear turn (Figures 2E and G), in agreement with a hearing loss involving specifically high-frequency regions during normal aging processes. In age-matched Cx30 ΔΔ animals, a marked OHC loss was observed, involving also apical and middle cochlear turns (Figures 2B, D, F, and G). That is to say, in the Cx30 ΔΔ group, in addition to cell damage in the basal cochlear region, cell count showed about 20% of OHC loss in the apical turn and about 30% in the basal middle turn, as compared to WT animals (Figure 2G). We estimated the number of IHCs in cochlear specimens and found that Cx30 ΔΔ animals showed a decreasing trend in all cochlear turns, although the difference between Cx30 ΔΔ and Cx30 WT was not significant (Figure 2H), suggesting that aging could be the dominant factor determining IHC damage.

Next, we focused on the neural cochlear compartment. SGNs are the first relay station of the afferent auditory pathway that conveys sensory information from the organ of Corti hair cells to the central nervous system and SGN degeneration is a hallmark of ARHL in mice (Bao and Ohlemiller, 2010). Therefore, we examined SGNs in Cx30 ΔΔ mice and we found a significant loss in apical and middle turn (Figures 2L–N) of the cochlea compared to WT age-matched animals (Figures 2I–K,O). The result of SGN loss with no IHC alterations is in line with published work showing that the loss of SGNs can greatly exceed the loss of IHCs following cochlear damage, and also in ARHL (Kujawa and Liberman, 2009; Wu et al., 2019). Altogether, these results confirm morphological damage and exacerbated presbycusis in Cx30 ΔΔ mice at 12 MoA.

### 3.2 Connexin 26 is downregulated in Cx30ΔΔ mice

To confirm the absence of Cx30 in the cochleae of Cx30 ΔΔ mice, we performed both immunofluorescence and western blotting studies in 12 MoA samples. Our data revealed no Cx30 expression in cochlear lysates of Cx30 ΔΔ mice, compared with Cx30 WT age-matched controls (Supplementary Figures S1A and B). Moreover, immunofluorescence analyses confirmed the absence of Cx30 labeling in Cx30ΔΔ mice (Supplementary Figures S1C and D).

**FIGURE 3:**
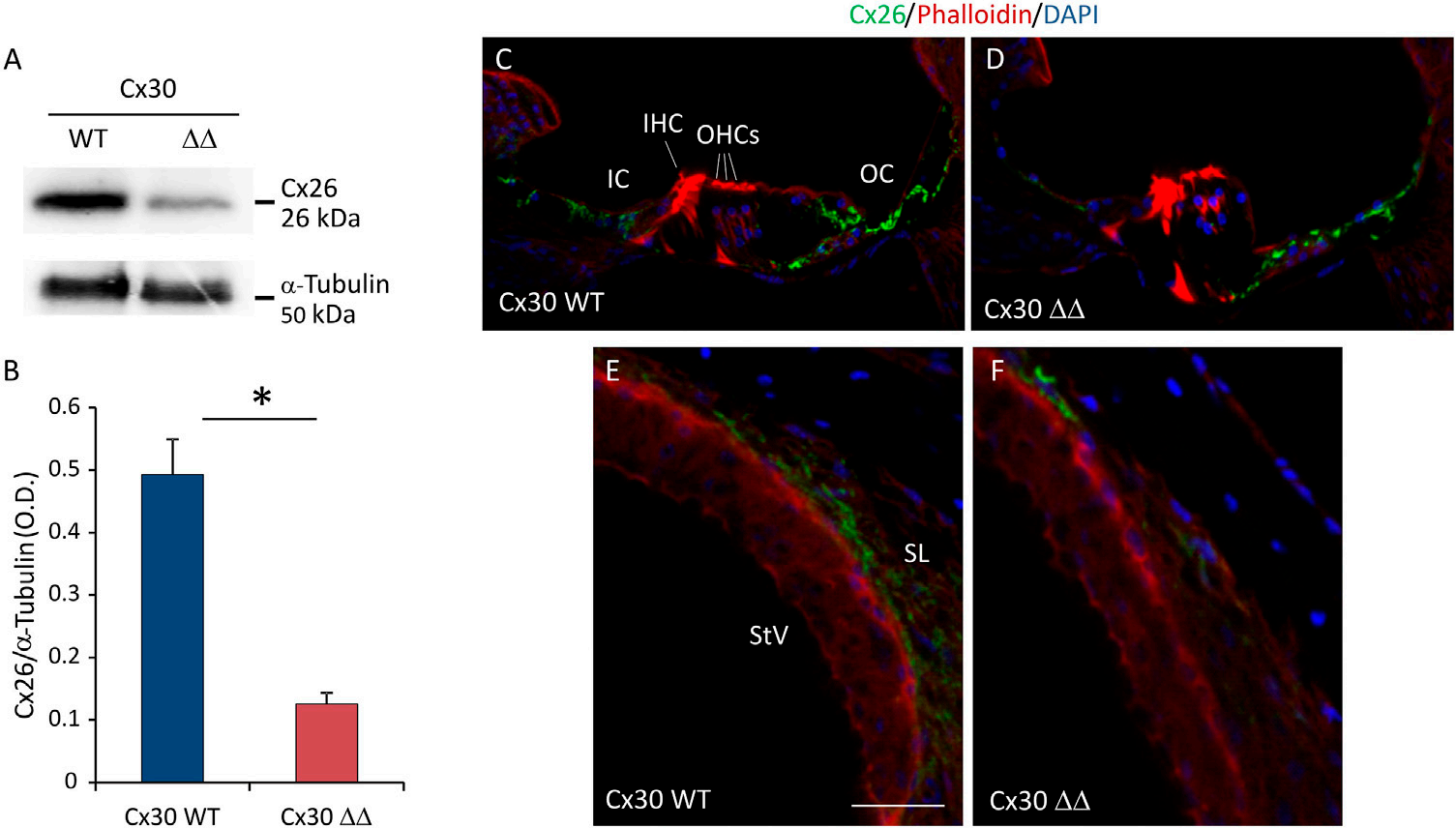
Cx26 expression in Cx30 ΔΔ-aging cochleae. **(A)** Representative western blot immunoreactive bands showing the expression of Cx26 in cochlear lysates from Cx30 WT and Cx30 ΔΔ animals at 12 months of age. **(B)** Histograms (mean ± S.E.M.) represent optical density values normalized to α-tubulin. N = 8 cochleae/group. **(C–F)** Immunofluorescence analysis for Cx26 expression in Cx30 WT and Cx30 ΔΔ organ of Corti **(C and D)** and stria vascularis and spiral ligament **(E and F)** from 12 months of age cochleae. IHC: inner hair cell; OHCs: outer hair cells; OS: outer sulcus; IC: inner sulcus; StV: stria vascularis; and SL: spiral ligament. Scale bar: 50 μm. N = 4 cochleae/group. Experiments were performed in triplicate. Asterisks indicate significant differences between groups from Student’s t-test (*p < 0.05).

Considering that, in young Cx30 ΔΔ mice the level of residual Cx26 protein is about 5 times higher than in other models of Cx30 KO and that it accounts for about 50% of total Cx26 expression (Boulay et al., 2013), we wondered if worsening of auditory threshold observed in Cx30 ΔΔ aged animals could be related to a decreased expression of Cx26. Of note, our immunofluorescence and western blot analyses showed a significant reduction of Cx26 expression in cochleae of Cx30 ΔΔ mice of 12 MoA compared with age-matched WT animals. In particular, western blot analyses revealed a residual expression of about 30% of Cx26 in Cx30 ΔΔ mice, with respect to WT total protein expression (Figures 3A and B). A strong reduction of Cx26 immunoreactivity was observed in the sensory epithelium of the organ of Corti in Cx30 ΔΔ aged animals (Figures 3C and D), as well as in the stria vascularis and spiral ligament where Cx26 signal was almost absent (Figures 3E and F). Note that Cx26 immunoreactivity was also weaker than expected in aging WT cochleae, specifically in the spiral ligament (Figure 3E), suggesting that Cx30 deletion can influence and probably exacerbate age-induced Cx26 downregulation.

### 3.3 Increased oxidative stress, inflammation, and vascular dysfunction in Cx30ΔΔ mice

Lateral wall and stria vascularis degeneration is the major contributor to the hearing loss associated with advancing age. Indeed, cochlear oxidative/inflammatory damage in conjunction with impaired blood flow is a typical feature of ARHL (Fetoni et al., 2011; Fetoni et al., 2022). Since Cx30 deletion exacerbates signs of ARHL in mice, we asked whether Cx30 ΔΔ animals show alterations in oxidative, inflammatory, and vascular marker expressions in the cochlea. To answer this question, we performed immunofluorescence and western blot analyses.

**FIGURE 4:**
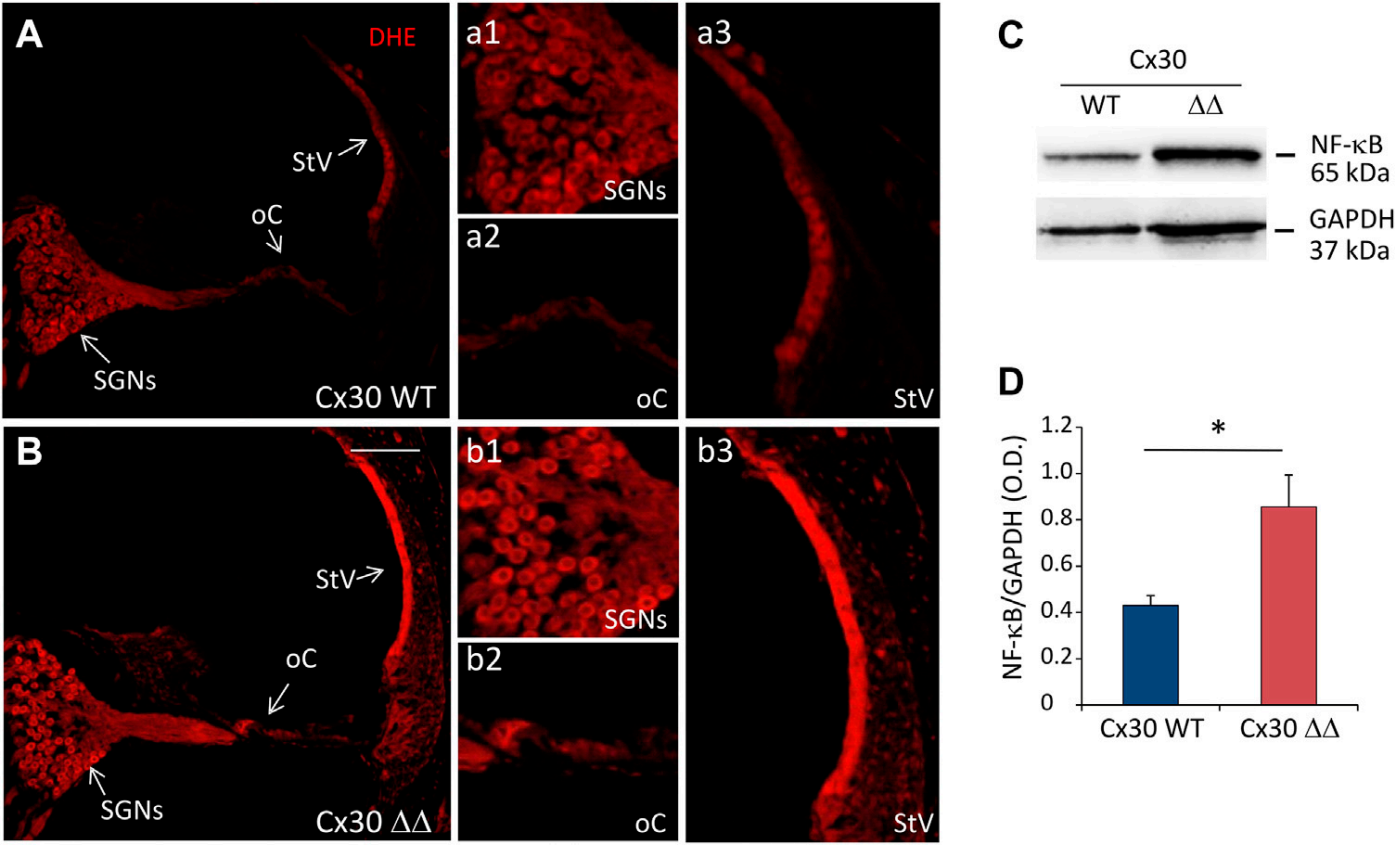
Increased oxidative stress and inflammation in the cochlea of Cx30 ΔΔ mice of 12 months of age. **(A and B)**: Representative images of cochlear cryosections from Cx30 WT **(A)** and Cx30 ΔΔ **(B)** mice of 12 months of age stained with DHE to analyze the ROS amount. N = 4 cochleae/group. High magnifications of spiral ganglion neurons (a1 and b1), the organ of Corti (a2 and b2), and stria vascularis (a3 and b3) are shown. **(C)**: Representative western blot immunoreactive bands quantifying the expression of NF-κB in cochlear lysates from Cx30 WT and Cx30 ΔΔ animals at 12 months of age. **(D)** Histograms (mean ± S.E.M.) represent optical density normalized to GAPDH. N = 8 cochleae/group. Asterisks indicate significant differences between groups from Student’s t-test (*p < 0.05). SGNs, spiral ganglion neurons; oC, organ of Corti; StV, stria vascularis.

To establish an initial correlation between Cx30 deletion and cochlear oxidative stress, DHE staining (to detect ROS generation) was performed in cochlear cryosections. Confocal fluorescence imaging indicate an increase of superoxide expression (red fluorescence) in cochlear samples of Cx30 ΔΔ animals compared to age-matched controls of 12 MoA (Figures 4A,B). In particular, the fluorescence increase was more evident in the stria vascularis (Figures 4a3,b3) than in the organ of Corti (Figures 4a2,b2) and SGNs (Figures 4a1,b1). Interestingly, the increase in oxidative stress was paralleled and associated with an enhancement of the inflammatory marker NF-κB. Indeed, western blot analysis showed a marked increase in NF-κB expression in cochlear lysates from Cx30ΔΔ-aged mice (Figures 4C,D).

**FIGURE 5:**
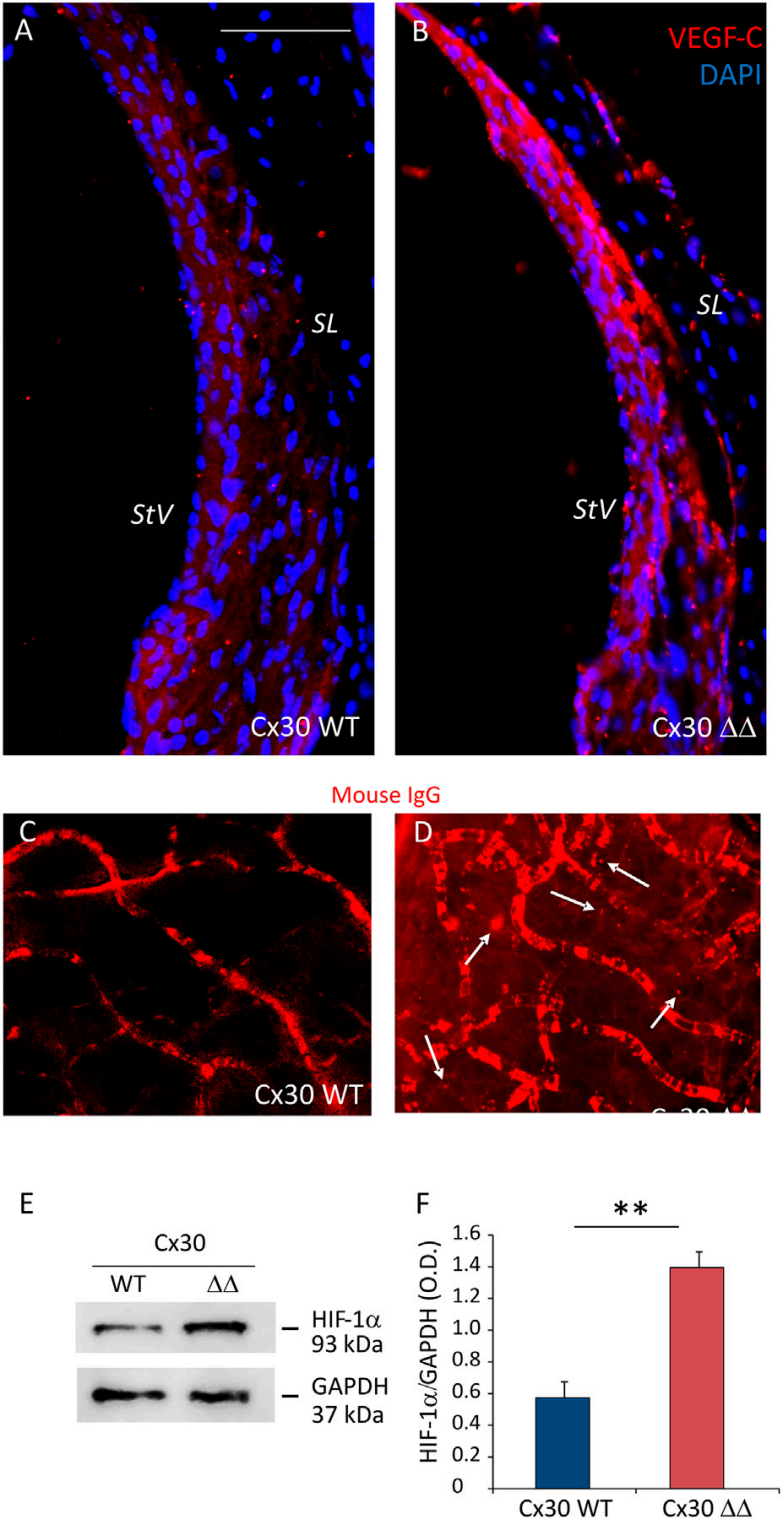
Deletion of Cx30 causes vascular dysfunction in the aging cochlea. **(A and B)**: Immunofluorescence analysis of VEGF-C (red fluorescence) in the stria vascularis of Cx30 WT and Cx30 ΔΔ cochlear samples. N = 4 cochleae/group. SL: spiral ligament and StV: stria vascularis. Scale bar: 100 μm. **(C and D)**: Representative images of explanted stria vascularis whole mounts from Cx30 WT **(C)** and Cx30 ΔΔ mice **(D)** stained with Alexa Fluor 546 mouse IgG. Arrows indicate mouse immunoglobulin fluorescence out of the vessels in the Cx30 ΔΔ sample. Scale bar: 30 μm. n = 3 cochleae/group. **(E)**: Western blot immunoreactive bands quantifying the expression of HIF-1α in cochlear lysates from Cx30 WT and Cx30 ΔΔ animals. **(F)**: Histograms (mean ± S.E.M.) represent optical density normalized to GAPDH. N = 8 cochleae/group. Experiments were performed in triplicate. Asterisks indicate significant differences between groups from Student’s t-test (**p < 0.01).

Moreover, since vascular dysfunction, in conjunction with oxidative stress and inflammation, is considered a common etiopathological marker for processes related to cochlear aging, we performed western blot and immunofluorescence analyses to evaluate vascular integrity in cochlear samples from Cx30 ΔΔ and WT mice. As a marker of vascular damage, VEGF expression often occurs in response to tissue ischemia/hypoxia through transcriptional upregulation by HIF-1α. Thus, we evaluated the expressions of both VEGF-C and HIF-1α in cochlear samples of 12 MoA mice. As shown by our western blot analysis in Figure 5, we found a significant increase of HIF-1α in cochlear lysates of Cx30 ΔΔ-aged mice, compared to WT (Figures 5E,F). In parallel, immunofluorescence experiments in stria vascularis cochlear cryosections revealed an increase in VEGF-C expression in Cx30 ΔΔ mice (Figures 5A,B). Consistent with the strong activation of HIF-1α, the increase of VEGF-C labeling in the stria vascularis of Cx30 ΔΔ animals suggests cochlear vascular dysfunction.

To confirm these data, we examined vascular integrity in stria vascularis whole mounts isolated from Cx30 WT and C30 ΔΔ cochleae of 12 MoA animals. As shown in Figure 5, there was no sign of extravasated mouse IgG in Cx30 WT samples (Figure 5C), whereas red fluorescence was detected outside stria vascularis capillaries in Cx30 ΔΔ samples (see arrows in Figure 5D), indicating an alteration of the endothelial barrier.

Altogether, these data indicate that Cx30 depletion in cochlear cells of aged mice can exacerbate cochlear degeneration in presbycusis, involving the interplay among oxidative stress, inflammation, and vascular damage.

## 4 Discussion

The aim of the present study was to evaluate the possible impact of global deletion of Cx30 on the onset and/or progression of age-related cochlear dysfunctions. Our data demonstrate that, in the KO mouse model of Cx30 (Cx30 ΔΔ), in which Cx30 was removed without perturbing the surrounding sequences, ARHL and cochlear dysfunction were exacerbated. Indeed, we found that: 1) ARHL was more severe in Cx30 ΔΔ mice at 12 MoA, compared to age-matched WT controls; 2) cochlear morphological damage induced by aging was exacerbated in Cx30 ΔΔ mice, and both hair cells and spiral ganglion neurons were affected; 3) deletion of Cx30 induced Cx26 downregulation during aging; and 4) the molecular mechanisms underlying Cx30-mediated exacerbation of cochlear-aging involve oxidative stress, inflammation, and vascular dysfunction.

To our knowledge, this is the first evidence of the role of Cx30 in exacerbating cochlear dysfunction during aging.

C57BL/6J mice, the background strain used in this study, is the most frequently used murine model of presbycusis (Fetoni et al., 2011). These animals show a progressive hearing loss, reflecting the major types of ARHL phenotypes proposed by Schuknecht (Schuknecht, 1955; Schuknecht, 1964; Schuknecht and Gacek, 1993). Consistent with literature data, our functional long-term evaluations showed that WT C57BL/6J mice (Cx30 WT) exhibited an increase in auditory thresholds for high frequencies (20–32 kHz) starting from 6 MoA. At 12 MoA, a severe hearing loss (>70 dB) was observed (Fetoni et al., 2011; Zhang et al., 2013). Of note, we monitored the hearing threshold in Cx30 ΔΔ mice and we found that deletion of Cx30 did not affect hearing in young and adult mice (at 2 and 6 MoA), confirming the results of previous studies reporting that Cx30 could be dispensable for hearing (Boulay et al., 2013). However, interestingly, despite the fact that the total deletion of Cx30 did not affect ABR thresholds in young and adult mice, at 12 MoA, the absence of Cx30 makes cochlear structures more susceptible to processes related to cochlear aging, exacerbating hearing loss and cellular damage (decreased number of hair cells and SGNs). Thus, our functional and morphological results demonstrate for the first time that Gjb6 deletion can be considered a genetic risk factor for ARHL.

It is known that cochlear cell senescence and degeneration have a multifactorial etiology, involving both environmental risk factors (such as noise or ototoxic drugs) and genetic predisposition (Ohlemiller et al., 2000; McFadden et al., 2001; Fetoni et al., 2011; Fortunato et al., 2016; Wang and Puel, 2020; Fetoni et al., 2022). Indeed, in a previous study, we found accelerated ARHL in an animal model of Gjb2 deletion, the gene of the GJ protein Cx26, whose mutations are a major cause for congenital nonsyndromic profound hearing loss (Fetoni et al., 2018). In particular, in the heterozygosis model (Gjb2^+/−^), partial deficiency of the Cx26 protein was a determinant for increased mitochondrial ROS production and early aging cochlear damage (Fetoni et al., 2018). On the basis of these findings, we wondered if Cx30 deletion, increasing cochlear susceptibility to aging processes, affects also Cx26 expression, involving oxidative stress mechanisms. In this study, we used a KO mouse model (Cx30 ΔΔ) in which, in contrast to the previous model (referred to as Cx30^−/−^ in the literature) (Teubner, 2003; Gentile et al., 2021), Cx30 was removed without perturbing the surrounding sequences. Thus, the level of residual Cx26 protein is 5 times higher in Cx30 ΔΔ than in Cx30^−/−^ mice (Boulay et al., 2013). To our surprise, our data showed a more pronounced reduction of Cx26 expression (about 30% of residual Cx26 expression) in the aged cochlea of Cx30 ΔΔ mice, suggesting that both GJ proteins are involved in aging processes and that the lack of Cx30 can affect Cx26 downregulation during aging. Our hypothesis is that the absence of Cx30, together with the onset and progression of aging processes, can lead to degradation or instability of GJs, exacerbating also Cx26 age-dependent downregulation. Indeed, in C57BL/6J mice, a downregulation of both connexins (Cx30 and Cx26) during aging (Tajima et al., 2020) and a significantly reduced expression of Cx26 in the spiral ligament in aged mice, with a disruption in GJ connections among fibrocytes, have been reported (Ichimiya et al., 2000).

Looking for a molecular mechanism, we focused on the crossroad among oxidative stress, inflammation, and vascular dysfunction, considering that these damaging factors are common pathological markers of aging processes both in physiological cochlear-aging and in the genetic model of ARHL (Someya et al., 2009; Fetoni et al., 2018; White et al., 2018; Wang and Puel, 2020; Fetoni et al., 2022). Moreover, at the same time, deletions of connexins have been related to oxidative-inflammatory processes (Fetoni et al., 2018; Hua et al., 2021), as well as to vascular dysfunction and reduced endocochlear potential (Cohen-Salmon et al., 2007; Gentile et al., 2021; Chen et al., 2022). Our data, demonstrating that the absence of Cx30 and the consequent downregulation of Cx26 are associated with increased ROS amount in Cx30 ΔΔ aged-cochleae, suggest that connexin hemichannels play a key role in protecting against oxidative stress during aging, probably allowing diffusion of antioxidant molecules to counteract redox imbalance. Indeed, it has been demonstrated that astrocytes contribute to neuronal detoxification from ROS by releasing endogenous antioxidants through plasma membrane hemichannels formed by Cx43 (Shih et al., 2003; Rana and Dringen 2007), and our previous results with Gjb2 ± mice indicate that cochlear nonsensory cells release the endogenous antioxidant glutathione through hemichannels that contain Cx26 protomers (Fetoni et al., 2018).

The increase of oxidative stress induced by connexins downregulation is, in turn, responsible for increased inflammation, as indicated by the enhancement of NF-κB expression in Cx30 ΔΔ cochleae. Indeed, cumulative evidence also indicates an interplay between ROS and inflammation in several cochlear injuries, as in noise trauma, ototoxicity, and ARHL (Fujioka et al., 2014; Haase and Prasad, 2016; Kalinec et al., 2017; Kurabi et al., 2017; Fetoni et al., 2019; Fetoni et al., 2021; Paciello et al., 2021). ROS interacts with the NF-κB signaling pathway in many ways and the transcription of NF-κB-dependent genes has been shown to influence the levels of ROS that can, in turn, influence NF-κB activity (Gloire et al., 2006; Morgan and Liu 2011; Blaser et al., 2016; Fetoni et al., 2019).

In short, it is known that oxidative stress can also induce vascular dysfunction. Indeed, increased production of free radicals can lead to the activation of VEGF, an endothelial growth factor responsible for vascular angiogenesis, remodeling, and maintenance of the blood–brain barrier (Sondell et al., 1999; Miller et al., 2003; Rosenstein et al., 2010; London and Gurgel, 2014). On the other hand, cochlear vascular dysfunctions, including vasoconstriction or alterations in cochlear blood flow, have been considered risk factors for ARHL (Lyu et al., 2020; Peixoto Pinheiro et al., 2021). Our data showed an increased expression of HIF-1α, which plays a key role in cellular hypoxic response also by modulating the upregulation of VEGF-C in aged Cx30 ΔΔ cochleae. This suggests that the combined vascular dysfunction associated with ARHL and genetic predisposition further exacerbate ARHL. Our hypothesis is that the increase of oxidative stress and cochlear redox imbalance caused by connexin downregulation can, in turn, cause stria vascularis dysfunction and vascular dysregulation, worsening cochlear-aging processes. Moreover, our results are consistent with the literature findings showing alterations in cochlear blood flow and extravasation in Cx30 KO animal models (Cohen-Salmon et al., 2007) and a crucial role of Cx30 in maintaining the endocochlear potential (Chen et al., 2022). Considering that cochlear vascular changes, vasoconstriction, or alterations in cochlear blood flow are well known risk factors for ARHL (Lyu et al., 2020; Peixoto Pinheiro et al., 2021), it is plausible that total deletion of Cx30, in conjunction with Cx26 downregulation, can increase cochlear susceptibility to vascular damage, exacerbating aging processes.

In conclusion, our data support the hypothesis that Cx30 is not dispensable for hearing, in particular, during aging processes. Indeed, deletion of GJB6, with the consequent Cx30 ablation, makes the cochlear structure more vulnerable to senescence, worsening presbycusis, influencing Cx26 expression, and, in turn, exacerbating oxidative stress, inflammation, and vascular dysfunctions during cochlear senescence.

## Data Availability

The raw data supporting the conclusion of this article will be made available by the authors, without undue reservation.
