# Supplementary material for: Connexin 30 deletion exacerbates cochlear senescence and age-related hearing loss
Source: Front Cell Dev Biol. 2022 Aug 9;10:950837. doi: 10.3389/fcell.2022.950837 (PMC9395607; doi:10.3389/fcell.2022.950837)
Supplement: Supplementary file 1 [file DataSheet1.docx]

Supplementary Material

##
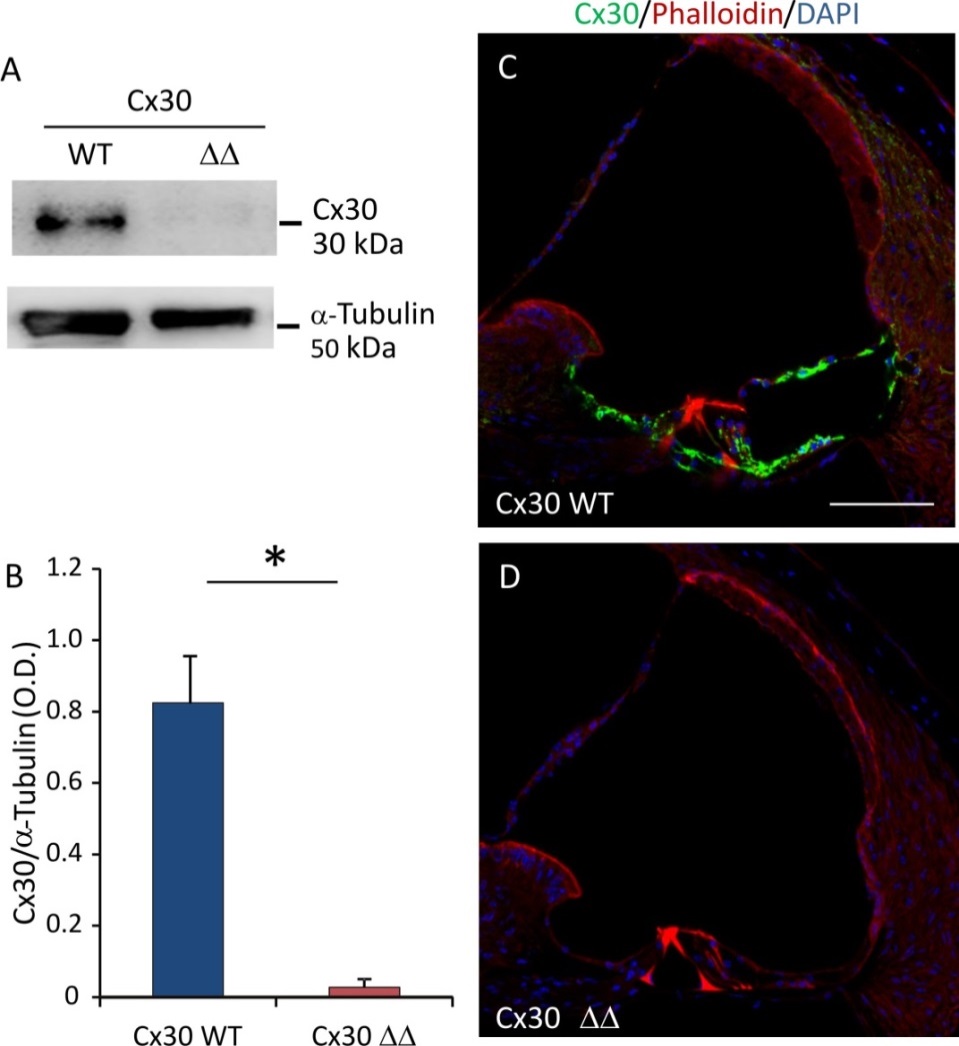
Supplementary Figures

**Supplementary Figure 1.** **Cx30 expression in Cx30 ΔΔ mice of 12 months of age.**

**A:** Representative western blot immunoreactive bands showing the expression of Cx30 in cochlear lysates from Cx30 WT and Cx30 ΔΔ animals at 12 months of age. **B:** Histograms (mean ± S.E.M.) represent optical density values normalized to α-tubulin. N= 8 cochleae/group. **C-D:** Immunofluorescence analysis for Cx30 expression in Cx30 WT and Cx30 ΔΔ cochleae from 12 months of age animals. Scale bar: 100 μm. N= 4 cochleae/group. Experiments were performed in triplicate. Asterisks indicate significant differences between groups from Student’s t-test (*p<0.05).

**Supplementary Table 1.** Statistical significant P values obtained comparing auditory brainstem response (ABR) thresholds for click stimuli and for tone bursts at 4, 8, 16, 24, 32 kHz obtained from Cx30 Wt and Cx30 ΔΔ animals at differ months of age (MoA).

| **Cx30 WT** | | | |
| --- | --- | --- | --- |
|  | **2 *vs* 6 MoA** | **6 *vs* 12 MoA** | **2 *vs* 12 MoA** |
| **Click** | P = 0.31 | P = 0.0008 | P = 0.012 |
| **4 kHz** | P = 0.099 | P = 0.0001 | P = 0.0001 |
| **8 kHz** | P = 0.017 | P = 0.0001 | P = 8.10 x10^-08^ |
| **16 kHz** | P = 0.319 | P = 0.036 | P = 2.34 x10^-05^ |
| **24 kHz** | P = 0.010 | P = 0.026 | P = 1.75 x10^-12^ |
| **32 kHz** | P = 4.89 x10^-05^ | P = 0.139 | P = 8.10 x10^-09^ |

| **Cx30 ΔΔ** | | | |
| --- | --- | --- | --- |
|  | **2 *vs* 6 MoA** | **6 *vs* 12 MoA** | **2 vs *12* MoA** |
| **Click** | P = 0.015 | P = 3.30x10^-05^ | P = 5.88 x10^-07^ |
| **4 kHz** | P = 0.286 | P = 0.000124546 | P = 1.38 x10^-06^ |
| **8 kHz** | P = 0.027 | P = 1.63 x10^-06^ | P = 1.27 x10^-08^ |
| **16 kHz** | P = 0.143 | P = 1.16 x10^-05^ | P = 3.19 x10^-09^ |
| **24 kHz** | P = 1.79368E-05 | P = 0.0003 | P = 1.31 x10^-15^ |
| **32 kHz** | P = 3.48195E-06 | P = 0.003 | P = 2.33 x10^-12^ |
